# Omission of adjuvant radiotherapy following breast-conserving surgery for elderly women with early-stage pure mucinous breast carcinoma

**DOI:** 10.1186/s13014-019-1394-x

**Published:** 2019-11-04

**Authors:** San-Gang Wu, Feng-Yan Li, Jun Wang, Chen-Lu Lian, Juan Zhou, Zhen-Yu He

**Affiliations:** 1 Department of Radiation Oncology, Cancer Hospital, the First Affiliated Hospital of Xiamen University, Teaching Hospital of Fujian Medical University, Xiamen, 361003 People’s Republic of China; 2 Department of Radiation Oncology, Sun Yat-sen University Cancer Center, State Key Laboratory of Oncology in South China, Collaborative Innovation Center of Cancer Medicine, Guangzhou, 510060 People’s Republic of China; 3 Department of Obstetrics and Gynecology, the First Affiliated Hospital of Xiamen University, Teaching Hospital of Fujian Medical University, Xiamen, People’s Republic of China

**Keywords:** Pure mucinous breast cancer, Radiotherapy, Elderly, Propensity score matching, Prognosis

## Abstract

**Background:**

We aimed to ascertain population-based practice patterns and survival outcomes of postoperative radiotherapy following breast conserving-surgery (BCS) in elderly women (aged ≥65 years) with early-stage pure mucinous breast carcinoma (PMBC).

**Methods:**

Patients aged ≥65 years diagnosed with T1–2N0 and hormone receptor-positive PMBC between 1990 and 2010 were identified from the Surveillance, Epidemiology, and End Results database. Binomial logistic regression, Kaplan-Meier method, Multivariate Cox proportional hazards models, and propensity score matching (PSM) were used for statistical analysis.

**Results:**

We enrolled 3416 patients, including 1225 (35.9%) and 2191 (64.1%) in the no-radiotherapy and radiotherapy cohorts, respectively. The percentage of patients receiving postoperative radiotherapy following BCS was significantly lower after 2004 (59.5% between 2004 and 2010), relative to that before 2004 (71.1% between 1990 and 2003; *P* < 0.001). Before PSM, the 10-year breast cancer-specific survival (BCSS) rates were 98.1 and 93.2% for patients with and without postoperative radiotherapy (log-rank test, *P* < 0.001), respectively. In the PSM cohort, receiving postoperative radiotherapy was associated with better BCSS rates, with 10-year BCSS rates of 97.6 and 94.5% in patients with and without postoperative radiotherapy, respectively (log-rank test, *P* = 0.001). Multivariate Cox proportional analysis indicated that receiving postoperative radiotherapy was an independent factor associated with better BCSS before (*P* < 0.001) and after PSM (*P* = 0.001), relative to those not receiving postoperative radiotherapy.

**Conclusions:**

This study shows a decreasing utilization of postoperative radiotherapy following BCS of elderly PMBC patients over time. However, postoperative radiotherapy following BCS should be administered for elderly women with PMBC owing to independent association with better survival.

## Background

Pure mucinous breast carcinoma (PMBC) is a rare type of breast carcinoma involving abundant extracellular mucin production, which accounts for approximately 1–6% of all cases of breast cancer [1, 2]. PMBC has distinct clinicopathological and molecular features, including higher estrogen receptor (ER) and progesterone receptor (PR) expression, greater likelihood of human epidermal growth factor receptor-2 (HER2)-negative status, lower grade, and lower risk of nodal metastasis [3–5], which all contribute to better outcomes compared to invasive ductal carcinoma (IDC); indeed, the 10-year disease-free survival rate is > 90% [6–12]. In addition, the median age of PMBC patients was 70 years, which was significantly greater than the age of those with other histological subtypes [11]. In clinical practice, the recommendation for adjuvant treatment of PMBC differs from that for the usual breast cancer histology [13].

Prior studies have found that breast-conserving surgery (BCS) is an appropriate surgical procedure for most patients with early-stage PMBC [11, 14]. In patients with invasive breast carcinoma, several prospective clinical trials have indicated that the omission of postoperative radiotherapy (RT) following BCS is safe and associated with an acceptable low risk of local recurrence and without a detriment to overall survival (OS) among female patients who are elderly (aged ≥50, 65, or 70 years), tumor size ≤5 cm (T1–2), node-negative (N0) disease, and ER-positive tumors [15–18]. However, none of these trials specified whether PMBC patients were enrolled. In addition, in the trials evaluating the omission of postoperative RT, endocrine treatment was mandatory and in fact, there were statistically significant differences in local control rates, even though the recurrence rates were very low in general [15–18]. Until today, there is no clear recommendation on the best management of elderly PMBC patients with low risk for local recurrence. In light of this, we used data from the large and contemporary Surveillance, Epidemiology, and End Results (SEER) program to determine population-based practice patterns and survival outcomes in PMBC patients receiving postoperative RT, particularly among the elderly population.

## Materials and methods

### Patients

Patients diagnosed with PMBC between 1990 and 2010 were included from the SEER program. The SEER program is a population-based database maintained by the National Cancer Institute that includes the de-identified information of cancer incidence, demographics, first course of treatment, and vital status of approximately 28% of the United States (US) population [19]. Patients who met the following inclusion criteria were included in this study: 1) women with PMBC and aged ≥65 years; 2) treated with or without adjuvant external beam RT following BCS; 3) stage T1–2N0 disease; 4) ER- and PR-positive disease; and 5) available data on race/ethnicity, tumor grade, and chemotherapy. We defined aged ≥65 years as the elderly because this cut-off age regularly used in gerontology [20, 21]. Patients with metastatic disease, RT prior to surgery, receiving non- external beam RT, without positive histology, and unknown sequence of surgery and RT were excluded. This study was exempt from the approval process of the Institutional Review Board as it used de-identified data from the SEER program.

### Measures

The following variables were included in this study: age at diagnosis, race/ethnicity, grade, tumor stage, and whether chemotherapy and postoperative RT was administrated. The primary end-point of the present study was breast cancer-specific survival (BCSS), which was defined as the time from diagnosis to death from breast cancer.

### Statistical analysis

Patient demographics, clinicopathological variables, and treatment variables were compared using the chi-squared test based on whether postoperative RT was administered. A 1:1 propensity score matching (PSM) method including 5 variables (age at diagnosis, grade, race/ethnicity, tumor stage, and chemotherapy) was used to create the matched cohorts to reduce any potential confounding in the retrospective studies [22, 23]. The predictors of postoperative RT administration were assessed using binomial logistic regression. The survival curves were plotted with the Kaplan-Meier method and compared using the log-rank test. Multivariate Cox proportional hazard models were used to investigate the independent prognostic indicators associated with BCSS. Statistical analyses were conducted using SPSS Statistical Software (version 22.0, IBM Corporation, Armonk, NY, USA), and a *P* value < 0.05 was considered to indicate statistical significance.

## Results

### Patient characteristics

**Fig. 1:**
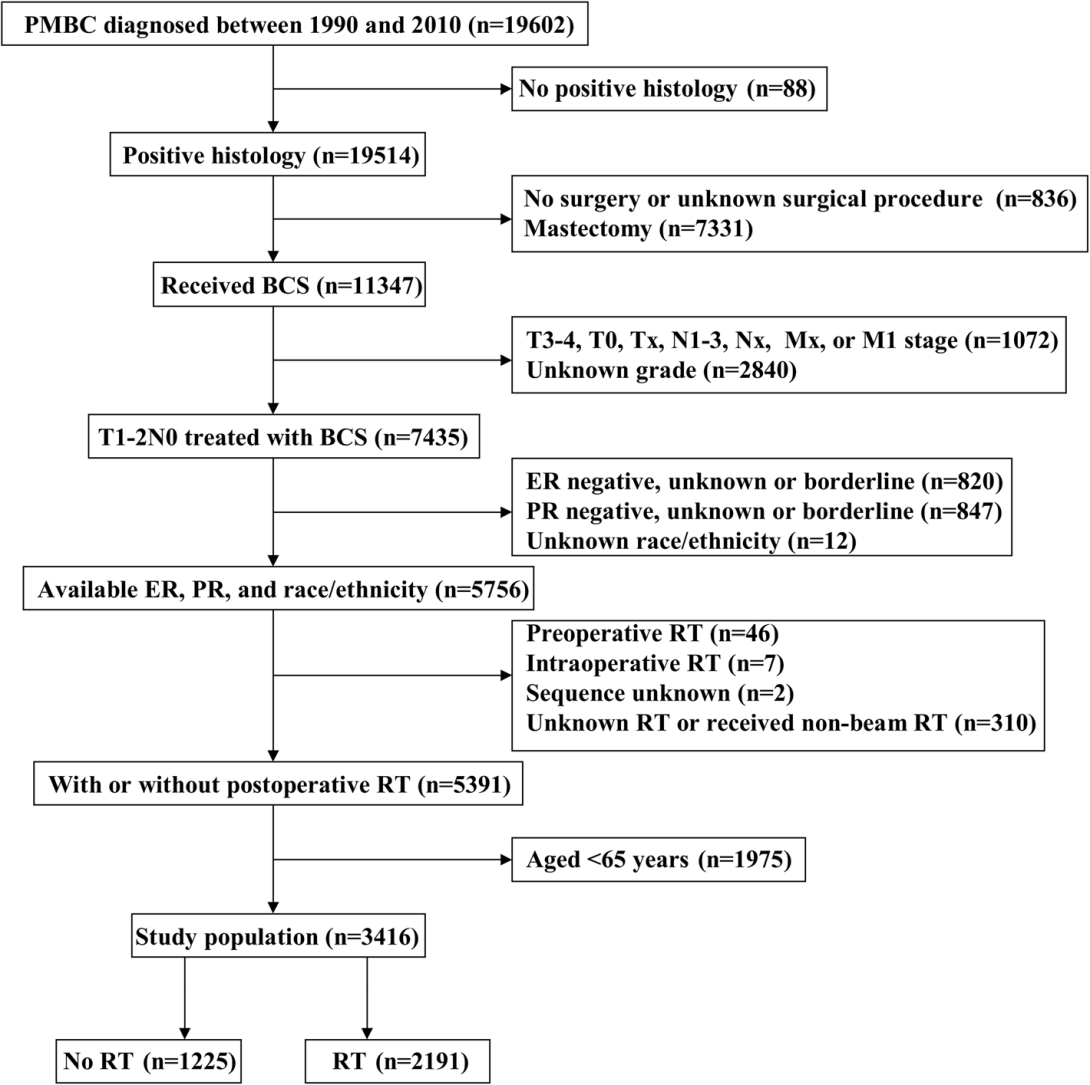
Patient selection flowchart of the study

We enrolled 3416 patients with a median age of 75 years (range, 65–99 years). Figure 1 shows the patient selection flowchart for the present study. Most patients were Non-Hispanic White (*n* = 2829, 82.8%), well-to-moderately differentiated disease (*n* = 3326, 97.4%), T1 stage disease (*n* = 1672, 83.4%), and did not receive chemotherapy (*n* = 3343, 97.9%). A total of 1225 (35.9%) and 2191 (64.1%) patients were assigned to the no-RT and RT cohorts, respectively. Patients with older age (*P* < 0.001), Non-Hispanic White (*P* = 0.039), T2 stage disease (*P* = 0.041), and no receipt of chemotherapy (*P* < 0.001) were more likely to undergo postoperative RT. In addition, a total of 1010 pairs of patients were completely matched using PSM. Table 1 lists the patient characteristics before and after PSM.

**Table 1:** Baseline characteristics of the two treatment cohorts before and after propensity score matching

Variables	Before PSM				After PSM			
	n	Non-RT (%)	RT (%)	P	n	Non-RT (%)	RT (%)	P
Age (years)								
65–69	681	123 (10.0)	558 (25.5)	< 0.001	244	122 (12.1)	122 (12.1)	1
70–74	840	210 (17.1)	630 (28.8)		420	210 (20.8)	210 (20.8)	
75–79	803	251 (20.5)	552 (25.2)		490	245 (24.3)	245 (24.3)	
> 79	1092	641 (52.3)	451 (20.6)		866	433 (42.9)	433 (42.9)	
Race/ethnicity								
Non-Hispanic White	2829	1035 (84.5)	1794 (81.9)	0.039	1694	847 (83.9)	847 (83.9)	1
Non-Hispanic Black	214	76 (6.2)	138 (6.3)		116	58 (5.7)	58 (5.7)	
Hispanic (All Races)	199	69 (5.6)	130 (5.9)		122	61 (6.0)	61 (6.0)	
Other	174	45 (3.7)	128 (5.9)		88	44 (4.4)	44 (4.4)	
Grade								
Well-differentiated	2279	837 (68.3)	1442 (65.8)	0.314	1358	679 (67.2)	679 (67.2)	1
Moderately differentiated	1047	356 (29.1)	691 (31.5)		622	311 (30.8)	311 (30.8)	
Poorly differentiated/undifferentiated	90	32 (2.6)	58 (2.6)		40	20 (2.0)	20 (2.0)	
Tumor stage								
T1	2848	1000 (81.6)	1848 (84.3)	0.041	1672	836 (82.8)	836 (82.8)	1
T2	568	225 (18.4)	343 (15.7)		348	174 (17.2)	174 (17.2)	
Chemotherapy								
No/unknown	3343	1213 (99.0)	2130 (97.2)	< 0.001	2000	1000 (99.0)	1000 (99.0)	1
Yes	73	12 (1.0)	61 (2.8)		20	10 (1.0)	10 (1.0)	

*PSM* propensity score matching; *RT* radiotherapy

### Trends of postoperative RT administration based on the period of diagnosis

**Fig. 2:**
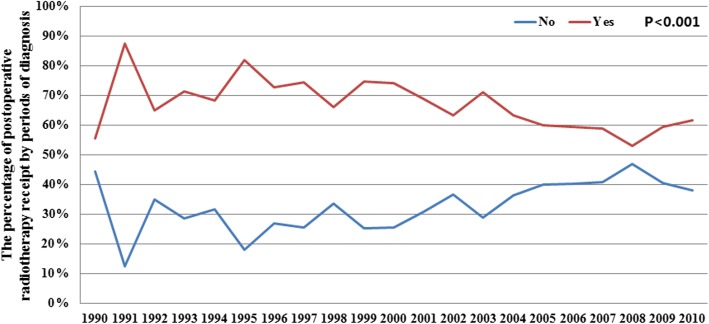
Change in the use of postoperative radiotherapy following breast-conserving surgery in patients with pure mucinous breast carcinoma, according to the period of diagnosis

Figure 2 lists the temporal trends of postoperative RT administration from 1990 to 2010. We found that the percentage of patients receiving postoperative RT was significantly lower after 2004 (*P* < 0.001). In particular, the percentage of patients receiving postoperative RT was 71.1% from 1990 to 2003, and decreased to 59.5% from 2004 to 2010 (*P* < 0.001).

### Independent predictive factors associated with receiving postoperative RT

Binomial regression analysis confirmed that younger age, moderately differentiated disease, and receiving chemotherapy were independently associated with receiving postoperative RT (Table 2).

**Table 2:** Independent predictive indicators of postoperative radiotherapy administration following breast-conserving surgery

Variables	OR	95% CI	P
Age (years)			
65–69	1		
70–74	0.669	0.521–0.860	0.002
75–79	0.492	0.385–0.630	< 0.001
> 79	0.154	0.124–0.197	< 0.001
Race/ethnicity			
Non-Hispanic White	1		
Non-Hispanic Black	0.917	0.673–1.249	0.582
Hispanic (All Races)	0.914	0.663–1.259	0.583
Other	1.432	0.990–2.069	0.056
Grade			
Well-differentiated	1		
Moderately differentiated	1.189	1.009–1.400	0.038
Poorly differentiated/undifferentiated	0.906	0.569–1.442	0.676
Tumor stage			
T1	1		
T2	0.931	0.762–1.136	0.480
Chemotherapy			
No/unknown	1		
Yes	2.219	1.158–4.251	0.016

*CI* confidence intervals; *OR* odds ratio

### Independent prognostic factors associated with BCSS

**Fig. 3:**
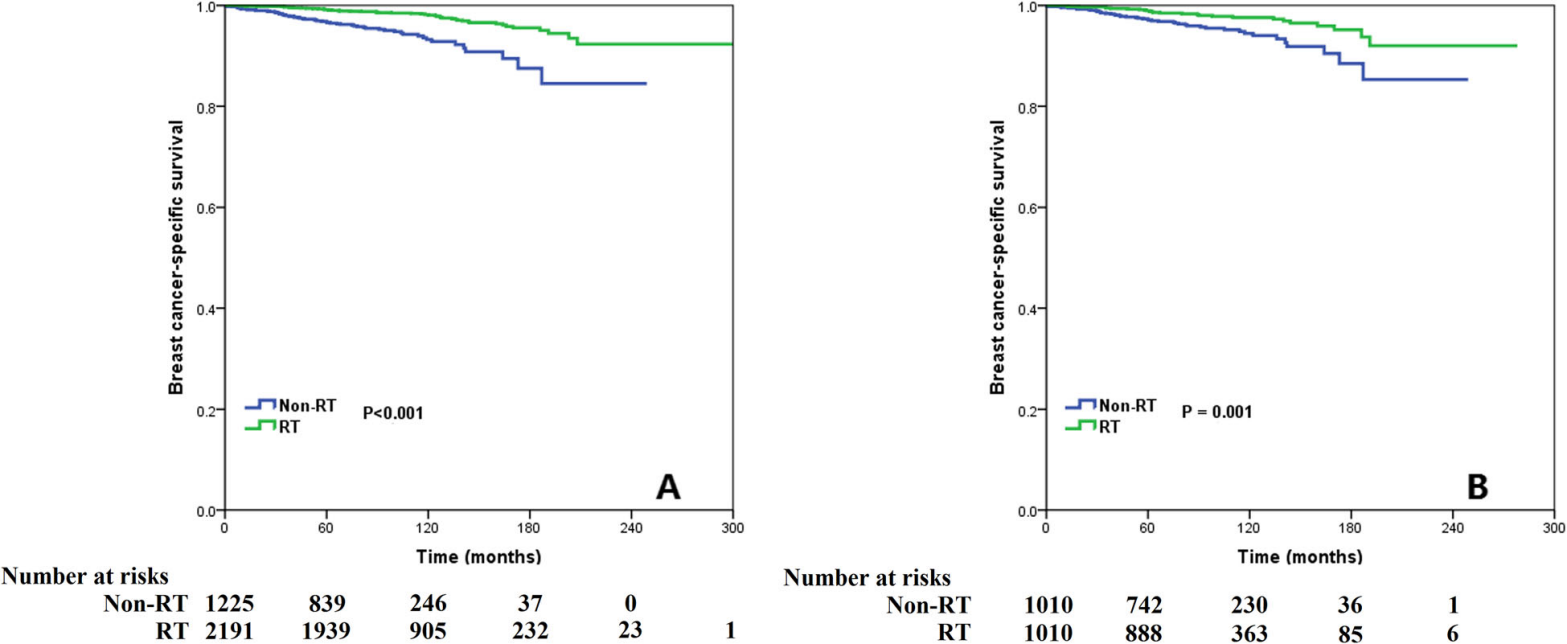
Comparison of breast cancer-specific survival between the non-radiotherapy and adjuvant radiotherapy cohorts before (**a**) and after (**b**) propensity score matching

Over a median follow-up period of 95 months (range, 0–304 months) in the non-PSM cohort, we found that 1591 (46.6%) patients died, including 110 (3.2%) patients who died of breast cancer. The 5- and 10-year BCSS rates were 98.3 and 96.6%, respectively. The 10-year BCSS rates were 98.1 and 93.2% in patients with and without postoperative RT, respectively (log-rank test, *P* < 0.001; Fig. 3a). Over a median follow-up duration of 90 months in the PSM cohort, we found that receiving postoperative RT was associated with a better BCSS; in particular, the 10-year BCSS rates were 97.6 and 94.5% in patients with and without postoperative RT after PSM, respectively (log-rank test, *P* = 0.001; Fig. 3b).

Multivariate Cox proportional analysis was used to determine the independent prognostic factors associated with BCSS (Table 3). After adjusting for age at diagnosis, tumor grade, race/ethnicity, tumor stage, and chemotherapy, we found that receiving postoperative RT was independently associated with better BCSS before (hazard ratio [HR]: 0.390, 95% confidence interval [CI]: 0.261–0.583, *P* < 0.001) and after PSM (HR: 0.445, 95%CI: 0.273–0.723, *P* = 0.001), compared to patients without postoperative RT.

**Table 3:** Multivariate analysis of the prognostic factors associated with breast cancer-specific survival before and after propensity score matching

Variables	Before PSM			After PSM		
	HR	95% CI	P	HR	95% CI	P
Age (years)						
65–69	1			1		
70–74	0.878	0.479–1.612	0.675	1.261	0.534–2.977	0.597
75–79	0.885	0.469–1.671	0.707	0.997	0.409–2.429	0.995
> 79	1.985	1.135–3.473	0.016	2.004	0.936–4.433	0.086
Race/ethnicity						
Non-Hispanic White	1			1		
Non-Hispanic Black	1.382	0.695–2.747	0.356	0.822	0.256–2.636	0.742
Hispanic (All Races)	0.676	0.248–1.842	0.444	0.713	0.223–2.288	0.570
Other	0.426	0.105–1.732	0.233	0.717	0.175–2.937	0.644
Grade						
Well-differentiated	1			1		
Moderately differentiated	1.195	0.797–1.791	0.388	1.211	0.736–1.922	0.452
Poorly differentiated/undifferentiated	2.429	1.111–5.311	0.026	2.256	0.693–7.350	0.177
Tumor stage						
T1	1			1		
T2	1.626	1.051–2.517	0.029	1.871	1.106–3.167	0.020
Chemotherapy						
No/unknown		1		1		
Yes	1.572	0.492–5.019	0.445	3.143	0.745–13.256	0.119
Radiotherapy						
No/unknown	1			1		
Yes	0.390	0.261–0.583	< 0.001	0.445	0.273–0.723	0.001

*CI* confidence intervals; HR, hazard ratio; *PSM* propensity score matching

## Discussion

In the present study, we aimed to assess the practice patterns and survival outcomes of postoperative RT following BCS in elderly PMBC patients, and found that the use of postoperative RT has decreased in recent years, even though it was associated with better BCSS before and after PSM. To our knowledge, this is the first study to assess the effect and administration of postoperative RT after BCS among elderly PMBC patients.

Although the present study only included patients aged ≥65 years, the patient characteristics in the selection flowchart showed that most patients had small tumor size, lower tumor grade, and ER- and PR-positive disease, consistent with that noted in previous studies [3–5]. In addition, most patients also had HER2-negative disease [3, 5]. Due to the less aggressive behavior of PMBC, the prognosis was excellent, with a 10-year BCSS rate of up to 96.6% in the present study. A retrospective analysis from a single institute or population-based study also showed an excellent BCSS of > 90% [3, 24].

Furthermore, the period of diagnosis impacted the decision of surgical procedure. A study by Sas-Korczyńska et al. included patients from 1952 to 2002 (*n* = 94) found that most patients were treated with mastectomy (95.7%) and only 4.3% received BCS [12]. Anan et al. also found that only 14.5% of patients received BCS between 1976 and 1998 (*n* = 76) [25]. However, 79.3, 51.3, and 64.1% of patients diagnosed in 1990–2010, 2014–2016, and 1983–2014, respectively, at 3 Chinese institutes still received mastectomy [10, 26, 27]. Several recent studies from the US showed that > 60% of patients were treated with BCS, and that this rate remained relatively constant between 1998 and 2018 [6, 11, 28]. The selection flowchart of our study also confirmed that approximately 60% of patients were treated with BCS, and that the prognosis in this patient subset was excellent. Our results also confirmed that BCS is an appropriate treatment for low-risk early-stage PMBC.

Previous clinical trials have sought to compare the outcomes between adjuvant endocrine treatment alone and adjuvant RT plus endocrine treatment following BCS in elderly patients with invasive breast carcinoma [15–18]; however, the histological subtypes of the enrolled patients were not recorded in those studies. As there are significant differences in the clinicopathological and molecular features between IDC and PMBC, it is unclear whether RT can be safely omitted in PMBC patients. However, in the present study, 64.1% of elderly patients were treated with RT, and the percentage of receiving postoperative RT significantly decreased from 2004 to 2010 (59.5%), relative to that from 1990 to 2003 (71.1%). The results of 2 prospective clinical trials were published in 2004, which showed that adjuvant RT plus tamoxifen was associated with better locoregional control but no significant difference in the rates of distant recurrence and OS compared to tamoxifen alone [15, 16], which could impact the treatment decision of postoperative RT after BCS in PMBC. Given the decrease in the use of postoperative RT in recent years, these findings could impact patient counseling, which is currently supported by findings from existing randomized studies conducted in IDC patients and may not be suitable due to the inherent lower aggressiveness of PMBC.

To our knowledge, no study has assessed the rates and patterns of local recurrence stratified based on whether postoperative RT was administered. Barkley et al. examined 111 PMBC patients (median age, 56 years), including 67% (*n* = 64) treated with BCS and 95.5% treated with RT following BCS. Over a median follow-up duration of 63 months, they found that 2 patients with stage T1 N0 and HR-positive disease developed local recurrence after adjuvant RT [26]. In addition, Vo et al. examined 61 patients (median age, 60 years) treated with BCS, including 90% treated with RT, and 3 patients were found to have developed local recurrence [14]. Gwark et al. assessed 471 patients from Korea (mean age, 46 years), including 65.6% who were treated with RT, and found a total of 34 relapses, including 10 patients of local recurrence [4]. Although we could not compare the effect of RT on local recurrence, based on the above results, the local recurrence rate of PMBC should be very low after postoperative RT. Hence, the administration of postoperative RT could potentially improve the outcomes of PMBC patients.

Limited studies were available to compare the outcomes of PMBC based on the administration of postoperative RT. A prior SEER study created a clinical nomogram to predict the outcomes of early PMBC [24]; however, the role of postoperative RT was not included in the nomogram. Gwark et al. found that RT was related to survival outcomes in univariate analysis, whereas no significant association with outcomes was observed in the multivariate analysis [4]; however, the surgical procedure was not specified in that study. Hence, we were unable to determine the role of RT on the outcomes on different surgical procedures. Another study from the SEER database that included 11,400 PMBC patients indicated that the addition of postoperative RT did not significantly improve OS; however, in that study as well, the surgical procedure was not analyzed [9]. To our knowledge, this is the first study to indicate the benefit of postoperative RT on BCSS in elderly PMBC patients, compared to those without postoperative RT. A large retrospective cohort study found that approximately 30% of PMBC patients did not receive postoperative RT following BCS [11], consistent with our findings in the elderly population. Thus, our findings show that postoperative RT should not be omitted following BCS in elderly PMBC patients.

Our study has certain limitations. First, inherent bias is possible in any retrospective study. Second, a centralized pathology review of the SEER database was lacking. Nevertheless, the survival rate of patients was similar to that of previous studies [6–12]. Thus, our study might still be representative of the relevant population. In addition, the details regarding endocrine therapy, chemotherapy regimen, radiotherapy dose, completeness of chemotherapy and radiotherapy, and the pattern of locoregional and distant recurrence were also not recorded in the SEER database. Finally, the percentage of patients receiving RT was under-reported in the SEER database [29]. Despite these limitations, our study offers new insights on the impact of postoperative RT on prognosis after BCS in PMBC patients. The SEER program provides us with a large sample size that can be used to analyze rare breast cancer histologies using robust statistical methods with sufficient power to draw conclusions. Thus, we believe that our findings will contribute to the current knowledge on the effect of postoperative RT following BCS in low-risk elderly PMBC patients.

## Conclusion

In conclusion, although the use of postoperative RT following BCS has been decreasing over time in elderly PMBC patients, we believe that postoperative RT may be beneficial in this patient subset; however, additional prospective studies are needed to confirm these results.

## Data Availability

Any request of data and material may be sent to the corresponding author.

## Abbreviations

BCS: Breast-conserving surgery
BCSS: Breast cancer-specific survival
CI: Confidence interval
CSS: Cause-specific survival
ER: Estrogen receptor
HER2: Human epidermal growth factor receptor-2
HR: Hazard ratio
IDC: Invasive ductal carcinoma
OS: Overall survival
PMBC: Pure mucinous breast carcinoma
PR: Progesterone receptor
PSM: Propensity score matching
RT: Radiotherapy
SEER: Surveillance, Epidemiology, and End Results
US: United States
